# Supplementary material for: Habitat preference, movements and growth of giant mottled eels, Anguilla marmorata, in a small subtropical Amami-Oshima Island river
Source: PeerJ. 2020 Nov 3;8:e10187. doi: 10.7717/peerj.10187 (PMC7646302; doi:10.7717/peerj.10187)
Supplement: Supplemental Information 1 [file peerj-08-10187-s001.pdf]

Supplemental Information

Habitat preference, movements and growth of Giant mottled eels, *Anguilla marmorata*, in a small subtropical Amami-Oshima island river

Hikaru Itakura[1,2*], and Ryoshiro Wakiya[3,4*]

[1]University of Maryland Center for Environmental Science,
Chesapeake Biological Laboratory, Solomons, Maryland, USA
[2]Graduate School of Science, Kobe University, Kobe, Hyogo, Japan
[3]Research and Development Initiative, Chuo University, Bunkyo-ku, Tokyo, Japan
[4]Present address: Atmosphere and Ocean Research Institute,
University of Tokyo, Kashiwa, Chiba, Japan

* Both authors contributed equally to this work.

Corresponding Author: itakurahikaru@gmail.com (H.I.)

List of contents

1) Supplemental Table

Table S1. Fish and crustacean species found in the study river.

2) Supplemental Figures

Figure S1. Distance from the river mouth vs eel density, depth, and velocity.

Figure S2. Eel density by each sampling time along distance from the river mouth.

Figure S3. TL or growth rate (GR) of eels in relation to their movement.

Figure S4. Relationships between eel GR and TL or study period.

Table S1. Fish and crustacean species found in the Oganeku River on Amami-Oshima Island, Japan, during the sampling surveys of the present study. The capture locations (i.e., downstream or upstream) of fish and crustacean species was defined depending on whether they were captured at more downstream or upstream locations relative to about 320 m from the river mouth where the riverscape greatly changed to higher-gradient upstream environments. Fishes and crustaceans were more abundant in downstream reaches of the river. The gobies (*Rhinogobius nagoyae* and *Sicyopterus japonicus*) and freshwater prawn (*Macrobrachium japonicum*) tended to be the more frequently encountered species.

Category	Species	Common name	Migratory type	Capture location
Fish	*Anguilla japonica*	Japanese eel	Diadromous	Downstream
Fish	*A. marmorata*	Giant mottled eel	Diadromous	Both
Fish	*Microphis leiaspis*	Pipefish	Diadromous	Downstream
Fish	*M. brachyurus*	Pipefish	Diadromous	Downstream
Fish	*Mugil cephalus*	Mullet	Diadromous	Downstream
Fish	*Chelon affinis*	Mullet	Diadromous	Downstream
Fish	*C. macrolepis*	Mullet	Diadromous	Downstream
Fish	*Kuhlia marginata*	Flagtail	Diadromous	Downstream
Fish	*K. rupestris*	Jungle perch	Diadromous	Downstream
Fish	*Acanthopagrus sivicolus*	Okinawa seabream	Diadromous	Downstream
Fish	*Eleotris acanthopoma*	Spinecheek gudgeon	Diadromous	Downstream
Fish	*E. fusca*	Dusky sleeper	Diadromous	Both
Fish	*E. melanosoma*	Broadhead sleeper	Diadromous	Downstream
Fish	*Yongeichthys criniger*	Goby	Diadromous	Downstream
Fish	*Redigobius bikolanus*	Speckled goby	Diadromous	Downstream
Fish	*Rhinogobius giurinus*	Goby	Diadromous	Downstream
Fish	*R. nagoyae*	Goby	Diadromous	Downstream
Fish	*R. brunneus*	Goby	Diadromous	Both
Fish	*R. yonezawai*	Goby	Diadromous	Upstream
Fish	*Tridentiger kuroiwae*	Goby	Diadromous	Downstream
Fish	*Luciogobius guttatus*	Flat-headed goby	Diadromous	Downstream
Fish	*Sicyopterus japonicus*	Goby	Diadromous	Both
Fish	*S. lagocephalus*	Red-tailed goby	Diadromous	Downstream

Fish	*Sicyopus zosterophorum*	Goby	Diadromous	Upstream
Crustacea	*Eriocheir japonica*	Japanese mitten crab	Diadromous	Both
Crustacea	*Macrobrachium lar*	Freshwater prawn	Diadromous	Upstream
Crustacea	*M. formosense*	Freshwater prawn	Diadromous	Downstream
Crustacea	*M. japonicum*	Freshwater prawn	Diadromous	Both
Crustacea	*M. gracilirostre*	Freshwater prawn	Diadromous	Upstream
Crustacea	*Caridina serratirostris*	Marsh shrimp	Diadromous	Downstream
Crustacea	*C. multidentata*	Marsh shrimp	Diadromous	Upstream
Crustacea	*C. typus*	Marsh shrimp	Diadromous	Both
Crustacea	*C. leucosticta*	Marsh shrimp	Diadromous	Downstream

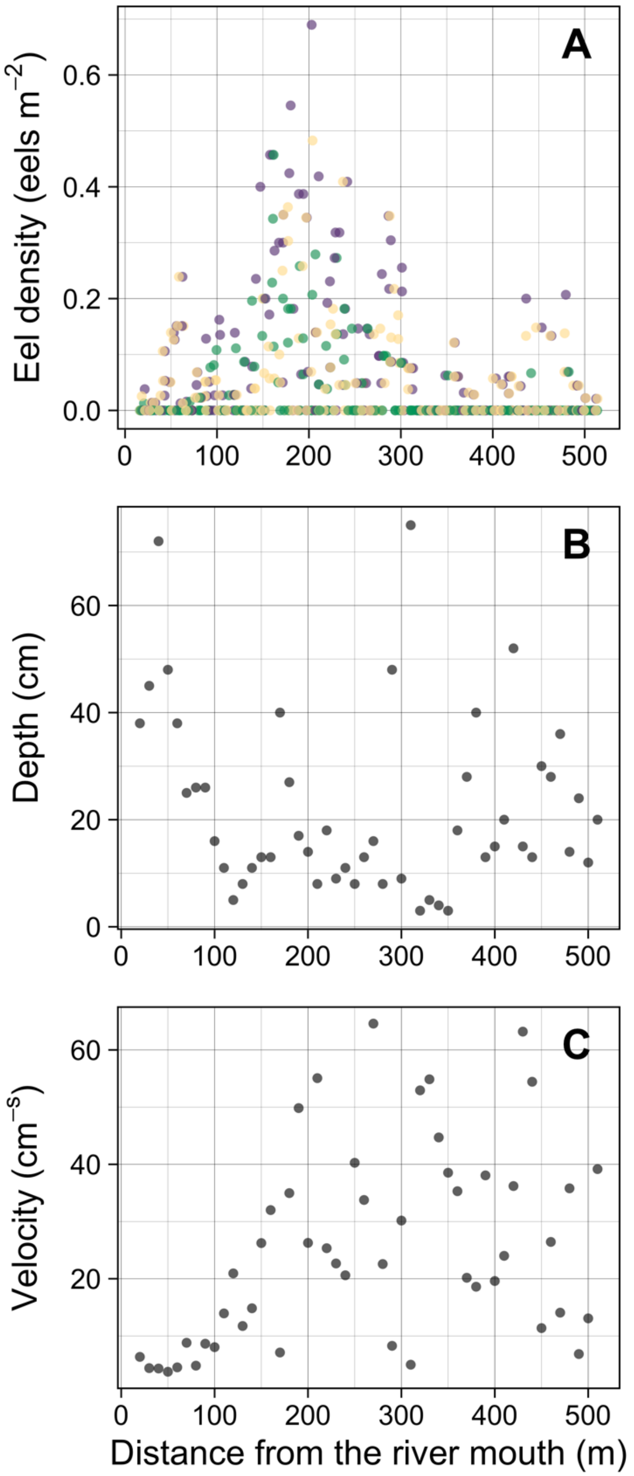

Figure S1. Relationships between distance from the river mouth and (A) *A. marmorata* density, (B) depth, and (C) velocity in sampling sections in the Oganeku River on Amami-Oshima Island, Japan.

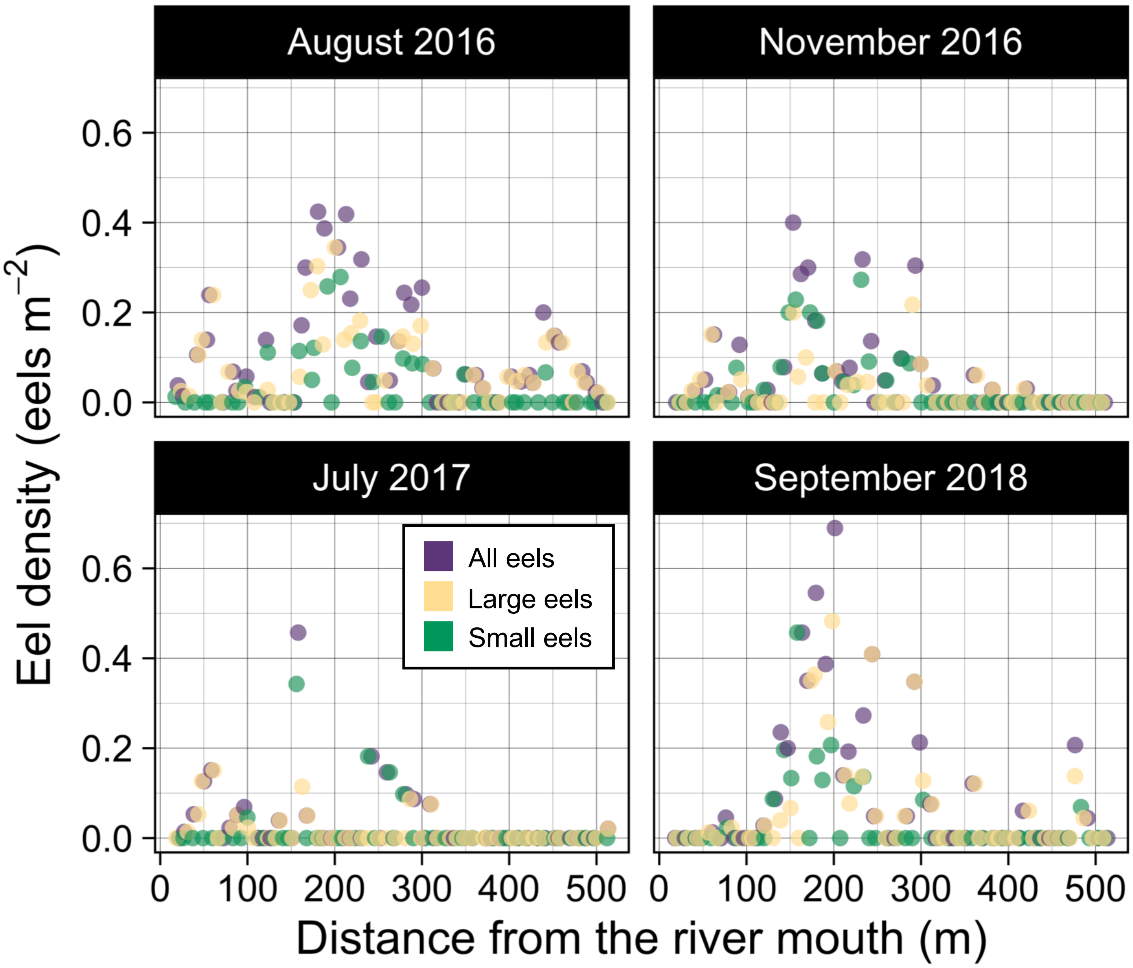

Figure S2. Density of *A. marmorata* in relation to the distance from the river mouth in the Oganeku River on Amami-Oshima Island, Japan during each of the 4 sampling periods.

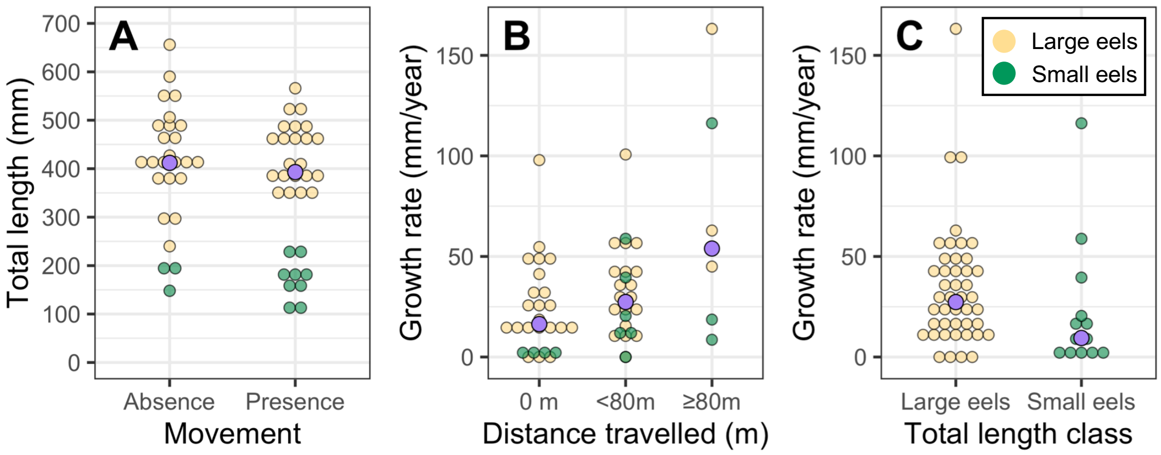

Figure S3. Total length or growth rate distribution of *A. marmorata* caught in the Oganeku River on Amami-Oshima Island, Japan, among (A) eels that moved or did not move, (B) eels that moved 0 m, <80 m, or moved ≥80 m, and (C) total length class. The purple circles denote median values.

The longest upstream movements were made by 3 small eels that moved from the 100 m section with gravel substrate and concrete and vegetated riverbanks to more upstream sections. There were 121 and 170 mm eels caught and tagged during the first sampling that were then captured 140 and 130 m upstream after 353 and 85 days at sizes of 139 and 172 mm (18.6, 8.6 mm/year GR), respectively. The longest movement was made by a 172 mm eel that was captured 424 days later and 380 m upstream at a size of 307 mm (116.2 mm/year GR).

The first two eels moved into gravel substrate and concrete and vegetated riverbanks habitats and the long movement eel moved to a boulder habitat section near the headwaters of the river. Three of the 4 fastest growing eels had made movements (380, 80, 40 m) and had inhabited mud, gravel, or boulder sediments and boulder riverbank habitats. These were 172, 417, and 566 mm eels that were caught and tagged during the first sampling period that were then captured after 424, 85 and 87 days at sizes of 307, 455 and 590 mm (116.2, 163.2, and 100.7 mm/year GR), respectively.

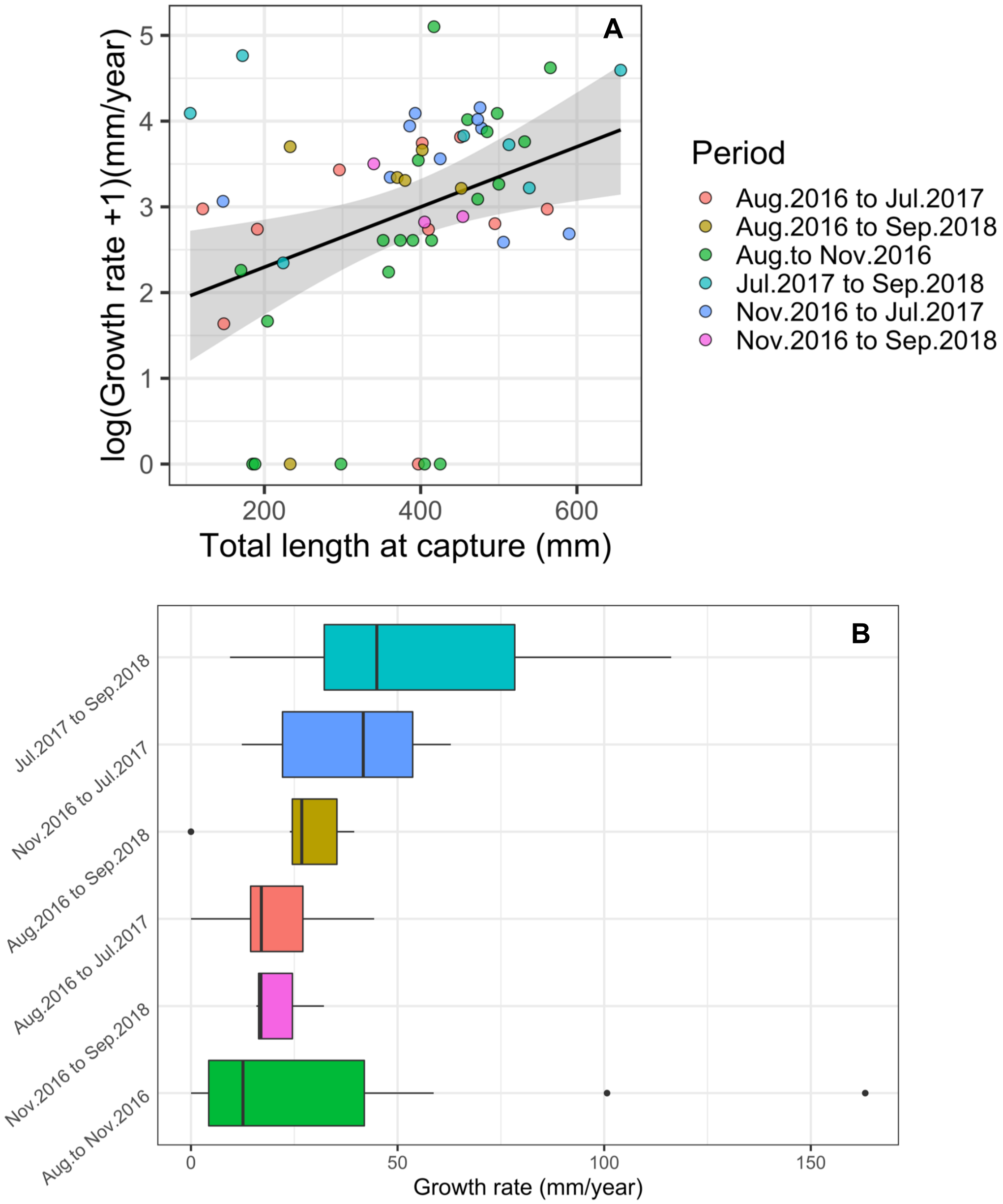

Figure S4. The relationship between total length at capture and growth rate (GR) of *A. marmorata* caught in the Oganeku River on Amami-Oshima Island, Japan, and the GR of eels during each study period between capture (marked) and recapture. The line and shaded area in (A) indicate the regression line and 95% intervals of the generalized linear model, respectively. In the boxplots (B), the middle lines indicate the median, the boxes represent the 0.25 and 0.75 quartiles, the whiskers are the values that are within 1.5 of the interquartile range, and the dots show outliers.
